# Asynchronous teledentistry program in two long‐term care facilities: Iowa's virtual dental home pilot project

**DOI:** 10.1111/scd.13074

**Published:** 2024-10-16

**Authors:** Jennifer E. Hartshorn, Pamela C. Nwachukwu, Tessa Heeren, McAllister Castelaz, Tessa Johnson, Chandler Pendleton, Paul Glassman, Steve M. Levy, Julie C. Reynolds

**Affiliations:** ^1^ Department of Preventive and Community Dentistry University of Iowa College of Dentistry Iowa City Iowa USA; ^2^ Health Research and Policy University of Iowa College of Public Health Iowa City Iowa USA; ^3^ Community Health Centers Southeastern Iowa Keokuk Iowa USA; ^4^ Division of Biostatistics and Computational Biology University of Iowa College of Dentistry Iowa City Iowa USA; ^5^ California Northstate University College of Dental Medicine Elk Grove California USA

**Keywords:** dental access, dental hygienists, geriatric, long‐term care, teledentistry, virtual dental home

## Abstract

**Introduction:**

Older adults living in long‐term care facilities (LTCFs) often have poor oral health and difficulty accessing dental services. The aim was to implement a virtual dental home (VDH) program for residents in two LTCFs utilizing asynchronous teledentistry techniques and mobile dental equipment.

**Methods:**

This pilot project was a 6‐month longitudinal cohort study in two LTCFs. Dental hygienists and dentists from a local community health care center utilized asynchronous teledentistry to provide diagnostic and preventive dental services onsite and to establish a dental home for additional comprehensive care services. Data were collected, including resident's demographics, oral health status, and dental services received. Descriptive data analyses, Wilcoxon signed rank, and McNemar tests were conducted.

**Results:**

Thirty‐four residents completed in‐person baseline oral exams and 16 residents completed the recall exams. Ninety‐two percent (*n* = 23) of dentate residents (n = 25) had untreated caries and 64% (*n* = 16) of dentate residents had at least one sextant of severe gingival inflammation. The median number of teeth with active untreated caries (*p* = .01) significantly decreased and arrested caries (*p* = .02) significantly increased from baseline.

**Conclusion:**

Iowa's VDH pilot project was successful in establishing a dental home using asynchronous teledentistry for residents in two LTCFs and providing resident access to preventive and disease control dental services.

## 1 INTRODUCTION

Older adults are the fastest growing segment of the U.S. population. Between 2022 and 2050, the proportion of the population aged 65+ is expected to grow in the U.S. from 17% to 23%, and from 10% to 16% globally.^1^, ^2^ With increasing age, comes greater prevalence of chronic diseases and disability.^3^ Due to this shift in age distribution, there is an expected increase in need for long‐term care options for older adults. The Centers for Medicare and Medicaid reported that in 2022, there were 15 000 long‐term care facilities (LTCFs) across the United States, serving 1.2 million residents.^4^

Patients in LTCFs are known to generally have poor oral health and substantial barriers to accessing dental care. Recent estimates of oral disease in LTCF residents indicate that 20%–51% have oral soft tissue lesions.^5^, ^6^, ^7^ —Five to 17% experience denture pain.^8^, ^9^, ^10^, ^11^ —Sixty‐six to 74% experience gingivitis,^8^, ^9^, ^12^ and 41%–79% have untreated caries.^8^, ^9^, ^10^, ^12^, ^13^, ^14^, ^15^, ^16^, ^17^ Barriers to dental care access among people living in LTCFs include the areas of medical complexity, transportation, mobility, cooperation, cost, lack of perceived need, and provider availability. ^18^, ^19^

One approach to reducing barriers to dental care for individuals in LTCF is the use of telehealth in dentistry. Telehealth refers to a broad variety of technologies and tactics to deliver virtual medical, health, and education services.^20^ Telehealth is not a specific service, but a collection of means to enhance care and education delivery.^20^ Teledentistry has been used to provide oral health care services to vulnerable populations and facilitate access to specialty care. Potential benefits of teledentistry include enabling/enhancing access to dental care, decreasing cost of care, and reducing oral health care disparities.^21^

One predominant model of teledentistry that has been utilized for over a decade to extend the reach of the dental clinic and reduce access barriers is the Virtual Dental Home (VDH).^22^ This model was developed by Dr Paul Glassman and colleagues in California to connect a dental hygienist located in a community setting (e.g., school, LTCF) with a dentist in an office in order to enable provision of diagnostic and preventive services for patients in the community setting. ^22^ In this model, the dental hygienist in the community setting gathers diagnostic data (e.g., radiographs, photographs) provides preventive services on site and sends the diagnostic information to the dentist. The in‐office dentist then reviews the data to complete diagnoses and treatment plan. If in‐office treatment is needed, the transportation required and the time in the dental office can then be used more efficiently.^22^

Older adults are often excluded from efficacy studies, leaving providers to extrapolate clinical decisions from studies in younger cohorts.^23^ Similarly, there are numerous reports describing teledentistry program implementation in pediatric populations and very few for older adults or nursing facility residents.^13^, ^21^, ^24^ A scoping review by Ben‐Omeran et al.^21^ found seven published studies with residents in LTCFs with only one evaluating the feasibility of developing remote treatment plans utilizing teledentistry and one evaluating soft tissue pathology.^21^, ^25^, ^26^ The remaining studies targeted oral health education, oral health quality of life, and potential cost savings.^21^, ^25^, ^27^, ^28^, ^29^ However, several authors have provided commentary indicating teledentistry could be implemented for nursing facility populations.^30^, ^31^, ^32^ Due to the scarcity of published research utilizing teledentistry in nursing facilities, the purpose of this study was to implement a VDH for nursing facility residents utilizing asynchronous teledentistry. This study describes the results of pilot project in Iowa that implemented a VDH program connecting residents in two LTCFs with a dental team at a Community Health Center (CHC) dental clinic. Evaluation activities included an examination of residents’ oral health status, as well as dental services received. The long‐term goals for this project are to improve residents’ regular access to diagnostic and preventive services onsite, establish a dental home for residents, and improve care efficiency by eliminating the need for transportation to the dental office for diagnostic/preventive visits.

## 2 MATERIALS AND METHODS

### 2.1 Study design

This pilot project was a 6‐month longitudinal cohort study that was conducted over a period of 2 years (2022–2023) in two LTCFs in conjunction with a local CHC and University based research team. The project was based on the VDH model utilizing store and forward teledentistry methodology (i.e., asynchronous teledentistry) to determine if patients should be seen by a dentist or can be maintained in the community setting.^22^ For this pilot project, the University‐based research team and a VDH consultant provided planning and implementation support and collected data to evaluate the program, while the CHC team administered the VDH program and provided patient direct care for residents of the LTCFs.

### 2.2 Facility and participant enrollment

This project partnered with two LTCFs located in the same county as the CHC dental clinic. The first was a 55‐bed nursing facility (LTCF1) and the second was an 84‐bed nursing facility (LTCF2) embedded within a retirement community.

The LTCFs helped CHC staff identify which residents were their own decision‐makers and which residents had designated Healthcare Powers of Attorney (POA). This project was approved by the University of Iowa Institutional Review Board (ID 202202245). All residents or their designated POAs were contacted via mail, phone, or in‐person and invited to enroll in both the dental program and research study. It was possible for residents/POAs to provide written informed consent to participate in the dental program with the CHC and deny participation in the research component of this study. The only exclusion criterion was residents expecting a short stay due to rehabilitation services. If a resident transitioned from short‐term rehabilitation services to residential care, then the resident or POA were contacted for enrollment.

### 2.3 VDH workflow

Upon enrollment into the program, the CHC dentist reviewed patients’ health records and approved patients to be scheduled with the DH. The Iowa VDH model utilized a CHC DH and dental assistant onsite with a mobile dental unit (Dntlworks ProCart II) and mobile dental chair (Dntlworks supreme aluminum chair) to provide services at the LTCFs. The DH recorded tooth notes, obtained dental radiographs utilizing a portable x‐ray unit (NOMAD Pro 2) and digital x‐ray sensor (Dexis), obtained extra‐oral photos utilizing an extra‐oral camera (Canon SX60), and obtained close‐up tooth photos using an intra‐oral camera (RealCloud HD1). The DH uploaded all clinical notes via remote desktop and saved radiographs and photos locally to a laptop to be uploaded to the patient's electronic dental record (EDR) the next day at the CHC. Once all data had been uploaded into the patient's EDR, the CHC dentist reviewed the chart to complete diagnoses and develop a treatment plan. The CHC dentist then called POA by phone or the DH conducted in‐person discussions to obtain consent for further treatment, including additional preventive services that could be provided at the LTCF [e.g., silver diamine fluoride (SDF) application] and/or additional treatment services requiring transportation to the CHC dental clinic. Of note, the state in which this project was conducted did not allow DHs to place interim therapeutic restorations (ITRs). Following completion of diagnostic and preventive services for all consenting residents, the mobile equipment was moved to the other LTCF, with plans to return every 6 months to continue periodic exams and preventive services.

### 2.4 Data collection and analyses

The REDCap (Research Electronic Data Capture) program was used by the research team to collect and manage study data.^33^ Data sources included EDR data from the CHC demonstrating services received, demographic and health history information from the LTCFs, and oral disease information collected via an in‐person oral examination conducted by a member of the research team. Following the completion of the asynchronous teledentistry exam by the CHC, one study examiner (JH) traveled the LTCF to conduct an in‐person oral exam of ∼30 min duration. Oral exams were performed with residents in the mobile dental chair using an overhead light from the examiner's loupes, toothbrush, interproximal brush, dental mirror, and explorer. No periodontal probing was performed during this study to avoid the need for antibiotic prophylaxis prior to the research exam. For research exams, removal of plaque from dentures and/or teeth was completed with a toothbrush and interproximal brush, if applicable, prior to data collection. Oral exams included assessment of oral soft tissue lesions, dentate status, gingival inflammation (modified gingival index) per sextant, visual and tactile examination for cavitated carious lesions patient cooperation, patient mobility, and urgency of treatment needs. Oral exams did not include evaluation of the radiographs obtained by the CHC. The research oral exams were conducted at baseline and 6‐month intervals following the CHC asynchronous teledentistry exams.

Analyses were performed using the Statistical Package for Social Sciences (SPSS) version 28.0 and R Core Team version 4.4.1. ^34, 35^ Descriptive analyses included residents’ demographic characteristics (Table 1), oral disease status, treatment urgency, mobility and cooperation (Table 2), specific dental caries measures (Table 3), and procedures provided by the CHC (Table 4). Wilcoxon signed rank or McNemar's tests were used to determine statistically significant differences in untreated caries, arrested caries, and gingival inflammation, between baseline and 6‐month exams. Wilcoxon signed rank tests were used to compare participants’ caries experience severity, number of teeth with active untreated caries, and number of teeth with arrested caries. McNemar's tests were used to compare participants’ presence of untreated active caries, arrested caries, and maximum gingival inflammation. *p*‐values < .05 were considered statistically significant. No adjustments were made for multiple comparisons.

**TABLE 1:** Demographic characteristics of study subjects at baseline and 6‐month follow‐up.

	Subjects at baseline (*n* = 39) *N* (%)	Subjects at 6‐month follow‐up (*n* = 16) *N* (%)
Age (years)		
Mean (SD)	79.6 (11.1)	84.6 (9.2)
Age group		
<65	4 (10.3%)	0 (0%)
65–74	9 (23.1%)	2 (12.5%)
75–84	10 (25.6%)	5 (31.2%)
85–94	14 (35.9%)	7 (43.8%)
95+	2 (5.1%)	2 (12.5%)
Gender		
Male	13 (33.3%)	6 (37.5%)
Female	26 (66.7%)	10 (62.5%)
Race		
White	34 (87.2%)	15 (93.7%)
Black/African American	2 (5.1%)	1 (6.3%)
Unreported	3 (7.7%)	0 (0%)
Dental insurance		
No insurance/Sliding fee	3 (7.7%)	1 (6.3%)
Medicaid	31 (79.5%)	12 (75.0%)
Private dental insurance	5 (12.8%)	3 (18.7%)
Decision‐making		
Self	23 (59.0%)	6 (37.5%)
Power of Attorney	16 (41.0%)	10 (62.5%)
Long‐term care facility		
LTCF 1	26 (66.7%)	7 (43.8%)
LTCF 2	13 (33.3%)	9 (56.2%)

**TABLE 2:** Oral health status of study subjects examined at baseline and 6‐month follow‐up.

	Subjects examined at baseline (*n* = 34) *N* (%)	Subjects examined at 6‐month follow‐up (*n* = 16) *N* (%)
Soft tissue lesions		
Yes	11 (32.4%)	3 (18.7%)
No	23 (67.6%)	13 (81.3%)
Soft tissue findings^a^		
Denture stomatitis	1 (2.9%)	0 (0%)
Ulcer	3 (8.8%)	0 (0%)
Abscess	2 (5.9%)	2 (12.5%)
Epulis fissuratum	1 (2.9%)	0 (0%)
Candidiasis	1 (2.9%)	0 (0%)
Other	4 (11.8%)	1 (6.3%)
Number of edentulous arches		
0	20 (58.8%)	13 (81.3%)
1	5 (14.7%)	2 (12.4%)
2	9 (26.5%)	1 (6.3%)
Number of teeth remaining		
Mean (SD)	15.7 (11.8)	18.6 (8.8)
Number of teeth remaining		
0	9 (26.5%)	1 (6.3%)
1–6	2 (5.9%)	1 (6.3%)
7–12	2 (5.9%)	2 (12.5%)
13–18	4 (11.8%)	3 (18.7%)
19–24	6 (17.6%)	5 (31.3%)
25–32	11 (32.3%)	4 (25.0%)
Treatment urgency		
No treatment needed	11 (32.4%)	2 (12.5%)
Treatment needed, not urgent	16 (47.1%)	12 (75.0%)
Treatment needed, urgent	7 (20.5%)	2 (12.5%)
Mode of transfer to dental chair		
Self‐transfer	10 (29.4%)	6 (37.4%)
Staff assistance	15 (44.1%)	7 (43.8%)
Mechanical lift	9 (26.5%)	3 (18.8%)
Cooperation for dental exam		
Very cooperative	28 (82.4%)	13 (81.3%)
Somewhat cooperative	4 (11.8%)	3 (18.7%)
Somewhat uncooperative	1 (2.9%)	0 (0%)
Very uncooperative	1 (2.9%)	0 (0%)

^a^ Percentages may not add up to 100% due to subjects being eligible for multiple categories.

**TABLE 3:** Prevalence and severity and comparisons of oral disease among dentate study subjects at baseline and 6‐month follow‐up.

	Dentate subjects examined at baseline (*n* = 25) *N* (%) or Mean (range)	Dentate subjects examined at 6‐month follow‐up (*n* = 15) *N* (%) or Mean (range)	*p* ^*^
Most severe level of gingival inflammation			
None	1 (4.0%)	0 (0%)	.9^**^
Mild	2 (8.0%)	1 (6.7%)	
Moderate	6 (24.0%)	5 (33.3%)	
Severe	16 (64.0%)	9 (60.0%)	
Caries experience (DMFT ≥ 1)			–
Yes	25 (100%)	15 (100%)	
No	0 (0%)	0 (0%)	
Caries experience severity (DMFT median, [mean], range)	20, [18.8], (5 ‐ 28)	21, [21.3], (14 – 28)	.11
Presence of untreated active caries			.6
Yes	23 (92.0%)	12 (80.0%)	
No	2 (8.0%)	3 (20.0%)	
Number of teeth with active untreated caries^*^ (median, [mean], range)	4, [5.3], (0–15)	2, [3.2], (0–10)	**.01**
Presence of at least one retained root tip			–
Yes	13 (52.0%)	7 (46.7%)	
No	12 (48.0%)	8 (53.3%)	
Number of retained root tips (median, [mean], range)	1, [1.4], (0 ‐ 10)	0, [1], (0 – 4)	–
Presence of arrested caries			.11
Yes	10 (40.0%)	11 (73.3%)	
No	15 (60.0%)	4 (26.7%)	
Number of teeth with arrested caries^*^ (median, [mean], range)	0, [0.6], (0 ‐ 3)	2, [2.2], (0 – 7)	**.02**

* Wilcoxon signed rank and McNemar's tests.

** Bivariate test for gingival inflammation used a dichotomized version of this variable (None/Mild/Moderate vs. Severe).

Bolded *p*‐values indicate statistical significance *p* < 0.05.

**TABLE 4:** Dental services received by study subjects provided by the CHC (*n* = 38).

	Services	Patients receiving services *N* (%)	Total number of services provided *N* (%^b^)
Diagnostic	Exams Periodic exam (D0120)	18 (47.4%)	25 (41.0%)
	Limited exam (D0140)	4 (10.5%)	5 (8.2%)
	Comprehensive exam (D0150)	28 (73.7%)	29 (47.5%)
	Re‐evaluation exam (D0170)	2 (5.3%)	2 (3.3%)
	Imaging		
	Comprehensive series (D0210)	20 (52.6%)	22 (15.3%)
	Intra‐oral Periapical (D0220)	13 (34.2%)	22 (15.3%)
	Periapical—additional film (D0230)	7 (18.4%)	21 (14.6%)
	Bitewing—single film (D0270, D0272, D0273, D0274)	5 (13.2%)	9 (6.2%)
	Panoramic film D0330)	2 (5.3%)	2 (1.4%)
	Oral/facial photographic image (D0350) 2D Intra or extraoral photo (D0703)	24 (63.2%) 21 (55.3%)	43 (29.8%) 25 (17.4%)
Preventive	Caries risk assessment (D0601, D0602, D0603)	31 (81.6)	55 (19.5%)
	Prophy (D1110)	22 (57.9%)	38 (13.5%)
	Fluoride varnish (D1206)	22 (57.9%)	43 (15.2%)
	Nutritional counseling (D1310)	23 (60.5%)	45 (16.0%)
	Oral hygiene instruction (D1330)	8 (21.1%)	43 (15.2%)
	SDF (D1354)	21 (55.3%)	58 (20.6%)
Restorative	Resin‐based composite (D2330, D2331, D2332, D2335, D2391, D2392, D2393 D2394)	6 (15.8%)	20 (87.0%)
	Protective restoration (D2940)	2 (5.3%)	3 (13.0%)
Periodontal	Scaling in the presence of gingival inflammation (D4346)	1 (2.6%)	1 (50.0%)
	Full mouth debridement (D4355)	1 (2.6%)	1 (50.0%)
Prosthodontics^a^	Complete denture (D5110, D5120)	3 (7.9%)	5 (83.3%)
	Partial denture (D5211, D5212)	1 (2.6%)	1 (16.7%)
Surgical	Extractions (D7140, D7210)	7 (18.4%)	20 (100.0%)
Teledentistry modality	Teledentistry (asynchronous) (D9996)	32 (84.2%)	60 (100.0%)

^a^ Prosthodontic services completed by the research team.

^b^ Percent of the services provided was calculated based on the total number of services within each subsection.

## 3 RESULTS

**FIGURE 1:**
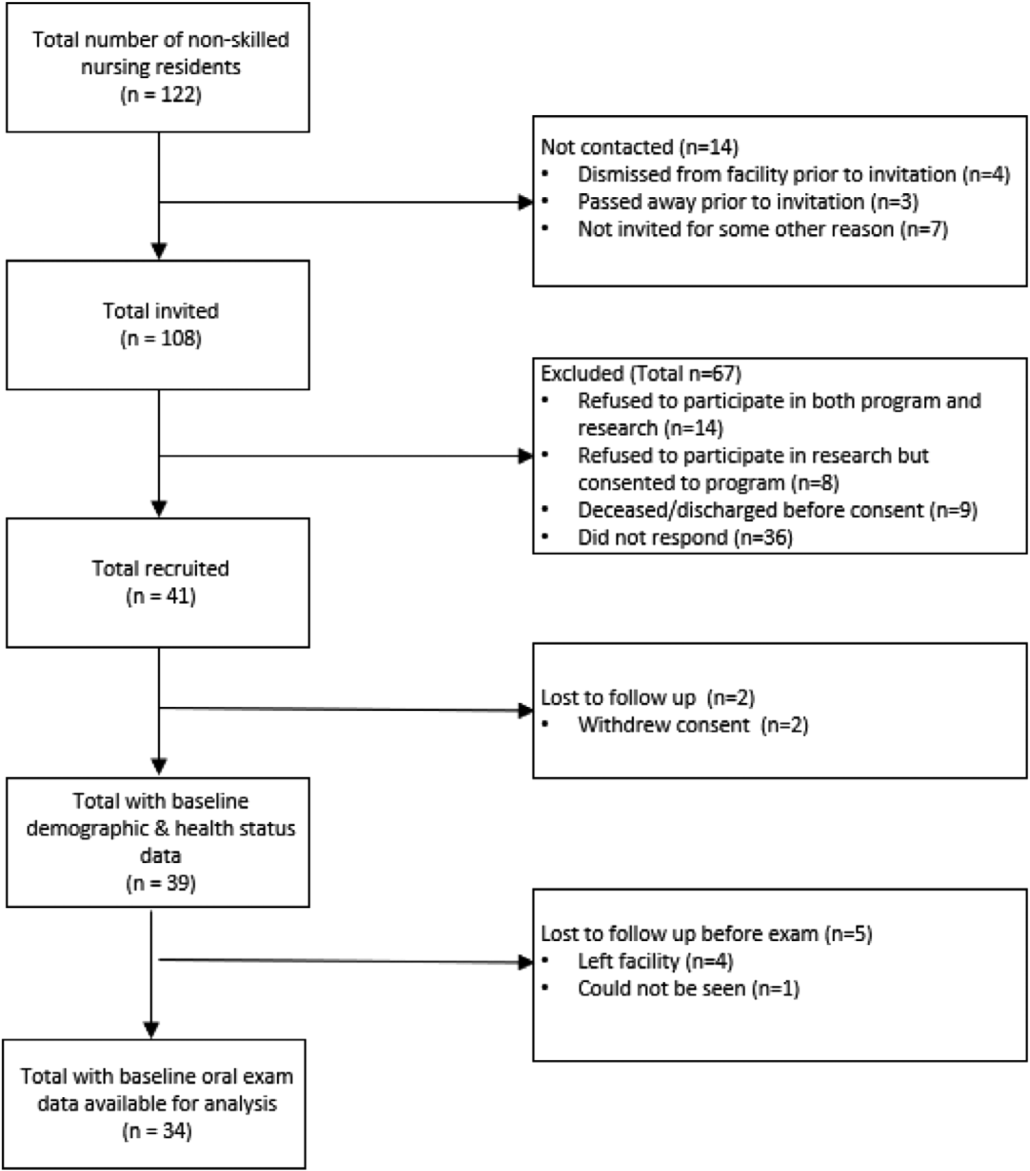
Subject flow diagram.

Throughout the project period, a total of 108 residents were invited to participate from both facilities (LTCF1 *n* = 59 and LTCF2 *n* = 49) and 36.1% (*n* = 39) elected to participate in both the VDH program and research component (Figure 1). LTCF1 (*n* = 26; 44.1%) enrolled more participants than LTCF2 (*n* = 13; 22.0%). The mean age of participants was 79.6 years at the initial exam. Study participants were predominantly female, white, Medicaid‐eligible, and residents who could make decisions for themselves (Table 1). The top six most common medical conditions present were hypertension 84.6% (*n* = 33), dementia 64.1% (*n* = 25), depression 59.0% (*n* = 23), hyperlipidemia 51.3% (*n* = 20), pain 46.2% (*n* = 18), and constipation 46.2% (*n* = 18). The top six most common medications prescribed to residents were acetaminophen 87.2% (*n* = 34), lisinopril 35.9% (*n* = 14), levothyroxine 35.9% (*n* = 14), aspirin 30.8% (*n* = 12), furosemide 28.2% (*n* = 11), and trazadone 28.2% (*n* = 11). Among the 39 residents who consented, 34 oral baseline exams and 16 six‐month recall exams were conducted. The reason so few recall exams were completed was due, in part, to several months of temporary closure of one of the LTCFs mid‐project period due to an extreme weather event.

With regard to resident oral health status documented at the in‐person research exams, the presence of soft tissue lesions and number of remaining teeth is detailed in Table 2. Among subjects examined at baseline (*n* = 34), 32% had soft tissue lesions, 27% were fully edentulous, and 21% had an urgent treatment need.

The prevalence and severity of resident oral health conditions, including gingival inflammation, root tips, and caries is detailed in Table 3. Among dentate subjects examined at baseline (*n* = 25), mean DMFT was approximately 19 teeth, 92% had untreated active caries with a mean of 5.3 teeth with active caries, and 52% had at least one retained root tip. From baseline to 6‐month follow‐up, the median number of teeth with active untreated caries declined significantly from 4 to 2 teeth (*p* = .01), and the median number of teeth with arrested caries significantly increased from 0 to 2 teeth (*p* = .02). Oral health measures for which there was no statistically significant change from baseline to 6 months included gingival inflammation, caries experience prevalence and severity, presence of untreated active caries, and presence of arrested caries.

Concerning dental services provided by the CHC clinic, 38 (97.4%) of the 39 consenting residents received services. Thirty‐two residents (84.2%) received an exam via teledentistry and 23 residents (*n* = 60.5%) received an on‐site dental cleaning coded as a dental prophylaxis, scaling in the presence of gingival inflammation, or full mouth debridement (Table 4). Following the teledentistry exams, five residents (14.7%) did not need follow‐up treatment with the dentist. Of the residents who required follow‐up care, three residents’ (8.8%) decision‐makers could not be reached by phone, five residents (14.7%) denied further care and requested to be monitored only, four residents (11.8%) consented to SDF treatment only, 10 residents (29.4%) consented to further disease management at the CHC clinic, three residents (8.8%) were referred to specialists due to case complexity, and four residents (11.8%) were referred to the university research team for removable prosthodontic services (no prosthodontic services were completed by the CHC). Of the 10 (28.6%) residents who consented to disease management at the CHC clinic, a total of six residents (17.4%) received follow‐up treatment services at the clinic. A summary of services received is shown in Table 4.

## 4 DISCUSSION

This pilot project focused on implementing the VDH model using mobile equipment and asynchronous teledentistry for residents in two LTCFs. Prior publications have reported that the most common uses of teledentistry were virtual asynchronous screening and diagnosis in the areas of pediatric dentistry and oral medicine.^24^ However, despite many recommendations to use teledentistry for older adults living in LTCFs, very few teledentistry studies among older adults have been published.^21^ Queyroux et al.^26^ found teledentistry can have a high level of accuracy compared to in‐person exams for diagnosing dental pathology in older adults living in LTCFs as the authors reported a 93.8% sensitivity, 94.2% specificity, and 6 (4.8%) false positives among 128 patients with dental pathology.^26^ Additionally, Mariño et al. evaluated the feasibility of nurses utilizing intra‐oral cameras to capture residents’ oral hard and soft tissues using video capture compared to face‐to‐face evaluations and reported high levels of resident satisfaction with teledentistry. However, this study did not report specific oral health findings of residents, with the exception of whether patients were dentate or edentulous. ^25^ Other articles published implementing teledentistry in LTCFs have focused on oral hygiene education for residents or direct care workers.^27^, ^28^, ^36^ Therefore, this pilot project is a novel design utilizing the VDH model to establish a dental home for LTCF residents, including offering preventive and diagnostic services on site and comprehensive follow‐up dental care services at the dental clinic, if needed.

The number of enrolled LTCF participants in this study was comparable or higher than in other studies evaluating the oral health of LTCF residents or offering onsite dental care.^5^, ^37^, ^38^, ^39^ For this study, it should be noted that LTCF1, which was located in a more rural location, enrolled more participants (*N* = 25) and had a higher percentage of participation among their residents (44.1%). This could be because residents had less access to dental care services due to the rural location. In contrast, LTCF2 was located in a town with three other dental offices in addition to the CHC dental clinic, which may account for the lower numbers of enrollment (*N* = 13) and lower percentage of participation among their residents (22%). In addition, it was possible for residents to participate in the dental program and decline research participation. Thus, these enrollment numbers may be an underestimation of the actual number of residents who obtained services from this program. Research in LTCFs can be particularly challenging, as residents may move, decline participation due to health status, or decline participation if the resident is likely to have limited cooperation for dental care. ^5^, ^18^, ^40^ Thus, resident participation in research may not fully represent all LTCF residents. Of note, it was also this pilot project plan to assess the degree of improvement of LTCF residents’ access to dental care as a result of this study. However, dental care utilization among individuals in LTCFs during the 2 years prior to the project period was extremely low due to the COVID‐19 pandemic. However, we noted that six residents (15.8%) were previous patients of the CHC and, therefore, received periodic exams instead of comprehensive exams as their initial exams with this VDH program. Despite challenges to enrollment and participation in services, it is well known that residents of LTCFs often have poor oral hygiene, oral pain, and high levels of oral disease,^41^ and it is important that residents living within LTCFs have access to oral health care services. Thus, it was not feasible to evaluate the degree of improvement in dental care access.

With regard to the evaluation of resident oral health during this study, it should be noted that few studies have evaluated the diagnostic accuracy of teledentistry among older adults. ^42^ For older adults, the accuracy of diagnosing oral dental pathology has been reported to have the potential to improve care quality and reduce costs and travel time required to see a specialist.^43^ With regard to caries, Pandey et al.^44^ evaluated intra‐oral photos captured with a smart phone in 18 patients aged 60–75 years and reported high sensitivity (> 91%), high specificity (> 98%), and almost perfect agreement (kappa value of 0.897–0.921) between examiners using asynchronous teledentistry for diagnosing caries compared to an in‐person clinical dental exam.^44^ In addition, Estai et al.^45^ reported a smartphone‐based mobile teledentistry model used by mid‐level dental providers showed potential in screening for dental caries as authors reported specificity ranging between 97% and 98% among examiners, sensitivity ranging from 60% to 68% among examiners, moderate to substantial agreement (kappa score 0.57–0.61) between in‐person and photographic exams, and almost perfect agreement between examiners completing a photographic exam (kappa score 0.89).^45^ While robust evidence of the diagnostic accuracy of teledentistry is not yet available, one study on a VDH project in California found that the CHC dentist was able to advise residents and decision‐makers whether or not to pursue additional dental services at the CHC clinic or services via referral. ^22^

The oral health conditions of LTCF residents in this study are similar to those from previously published studies. This study found 32.4% of participants did not need any treatment at baseline and 67.6% needed dental care. This is similar to study results from Gerritsen et al.,^40^ which found 73% of residents were in need of dental care. In addition, our study found 32.4% of participants had soft tissue lesions at baseline, which is similar to previous studies that reported from 20% to 51% of LTCF residents had soft tissue lesions.^5^, ^6^, ^7^ For gingival inflammation, our study found 64.0% of participants had at least one sextant of severe gingival inflammation and 88.0% had moderate or severe levels of gingival inflammation. These are relatively high levels of gingival inflammation compared to previous studies that reported 66%–74% of residents in LTCFs experienced gingivitis.^8^, ^9^, ^12^ For untreated caries, 92.0% of dentate residents had untreated carious lesions present at baseline. This is higher than previously published studies with 41%–79% of dentate residents of LTCFs having untreated carious lesions.^8^, ^9^, ^10^, ^12^, ^13^, ^14^, ^15^, ^16^, ^17^

While several studies reported on the poor oral health conditions of LTCF residents, very little research has been published regarding dental service utilization by LTCF residents, and what is published generally is a retrospective record review for at least one dental service on record^38^, ^39^, ^46^ or is a survey of patients and family.^47^, ^48^ However, one thesis reported on dental service utilization within LTCFs in which comprehensive dental care was offered on‐site to all residents at no cost. The study found that 84% of residents utilized dental services, including diagnostic (81.3%), preventive (72.2%), restorative (8.7%), surgical (11.9%), and prosthodontic (18.2%) services.^49^ However, this report did not include the resident's oral health conditions, so it is unclear how the need for services compared to utilization. Even our VDH program's utilization of services results should be interpreted with caution, as one facility experienced transportation issues delaying access to treatment services and many residents were lost to follow‐up with the temporary closure of a facility, causing residents to relocate to some distant communities and further preventing access to follow‐up care with this project. If others are to implement new programs utilizing the VDH model or mobile dentistry, it is important to not only understand the level of oral disease present in LTCF, but anticipate the utilization of services by LTCF residents which can affect a dental program's financing and staffing.

Another limitation to this study is that 82.4% of residents were very cooperative for their research exams, thus the oral health and services provided may be biased if additional patients with limited cooperation did not enroll or did not participate in the research portion of this project. The CHC did offer dental services utilizing minimal sedation or anxiolysis. If the CHC was unable to achieve the desired level of cooperation utilizing minimal sedation, patients are referred for care (8.8%). Thus, while the VDH model may not be able to provide comprehensive care to all patients in a LTCF, the VDH model still offered an entry into the dental care system.

There were several challenges regarding implementation of the pilot program in addition to the COVID‐19 pandemic delays. LTCF1 closed for several months, severely limiting the number of recall exams performed. Therefore, the recall examination data have limited generalizability when compared to the initial exam data. In addition, the CHC experienced staffing turnover during the project period, requiring new staff to be hired and trained to provide care in a LTCF using mobile dental equipment and teledentistry. While the CHC was the dental home for the residents, several residents needed specialized dental services requiring a referral for treatment which was not recorded as part of this project.

## 5 CONCLUSION

Iowa's VDH pilot project is a novel design utilizing the VDH model to establish a dental home for LTCF residents while offering diagnostic and preventive services on‐site and comprehensive services at the dental clinic. This program aimed to reduce dental care barriers and improve access to care for LTCF residents. Residents who participated in this project were found to have high levels of oral disease and benefited from access to onsite diagnostic and preventive services with the option of comprehensive dental care. Residents with untreated active caries and arrested caries significantly improved from baseline to 6 months. Future research should aim to quantify the degree of improvement in dental care access associated with VDH models, examine the degree of diagnostic accuracy when utilizing asynchronous teledentistry to conduct remote dental exams among LTCF populations, and ultimately assess improvements in oral health status and oral health‐related quality of life. Overall, the VDH model utilizing asynchronous teledentistry methods appears to be a viable model that can improve access to care for long‐term care facility residents with a high disease burden.

## CONFLICT OF INTEREST STATEMENT

Authors do not have any conflicts of interest to report.

## ETHICS STATEMENT

This project was approved by the University of Iowa Institutional Review Board (IRB ID #202202245).

## References

[1] Mather M SP . Fact Sheet: Aging in the United States. In: Bureau PR, editor; 2024.

[2] Nations U Global Issues: Ageing . Accessed March 8, 2024.

[3] ChildStats.gov Share of old age population (65 years and older) in the total U.S. population from 1950 to 2050. In Statista 2021. Accessed August 28, 2023. https://www.statista.com/statistics/457822/share‐of‐old‐age‐population‐in‐the‐total‐us‐population/

[4] Foundation KF KFF analysis of CMS Care Compare data: Total Number of Residents in Certified Nursing Facilities. 2022. Accessed August 16 2023. https://www.kff.org/other/state‐indicator/number‐of‐nursing‐facility‐residents/?currentTimeframe=0&sortModel=%7B%22colId%22:%22Location%22,%22sort%22:%22asc%22%7D

[5] Marchini L , Recker E , Hartshorn J , et al. Iowa nursing facility oral hygiene (INFOH) intervention: a clinical and microbiological pilot randomized trial. Spec Care Dentist. 2018;38(6):345‐355. 30194737 10.1111/scd.12327 PMC6246798

[6] Gerritsen PF , van der Bilt A , Cune MS , Schrijvers AJ , de Putter C . Integrated versus incidental dental care in nursing homes. Spec Care Dentist. 2013;33(5):227‐231. 23980555 10.1111/j.1754-4505.2012.00317.x

[7] Pietrokovski J , Levy F , Azuelos J , et al. Oral findings in elderly nursing home residents in selected countries. 2. Soft tissue lesions and denture wearing habits. Gerodontology. 1990;9(3):75‐81. 2133461 10.1111/j.1741-2358.1990.tb00262.x

[8] Hoben M , Clarke A , Huynh KT , et al. Barriers and facilitators in providing oral care to nursing home residents, from the perspective of care aides: a systematic review and meta‐analysis. Int J Nurs Stud. 2017;73:34‐51. 28531550 10.1016/j.ijnurstu.2017.05.003

[9] Matthews DC , Clovis JB , Brillant MG , et al. Oral health status of long‐term care residents‐a vulnerable population. J Can Dent Assoc. 2012;78:c3. 22364866

[10] Porter J , Ntouva A , Read A , et al. The impact of oral health on the quality of life of nursing home residents. Health Qual Life Outcomes. 2015;13:102. 26169066 10.1186/s12955-015-0300-y PMC4501060

[11] Adegbembo AO , Leake JL , Main PA , Lawrence HL , Chipman ML . The effect of dental insurance on the ranking of dental treatment needs in older residents of Durham Region's homes for the aged. J Can Dent Assoc. 2002;68(7):412. 12119091

[12] Pattrick DL MT , Bigby JA , Auerbach J , Mullen J , Johnson DE , Bethel LA . The Commonwealth's high‐risk senior population: Results and Recommendations from 2009 statewide oral health assessment. 2010. In: Massachusetts Department of Public Health OoOH. editor.

[13] Arpin S , Brodeur JM , Corbeil P . Dental caries, problems perceived and use of services among institutionalized elderly in 3 regions of Quebec. Canada J Can Dent Assoc. 2008;74(9):807. 19000464

[14] Chalmers JM , Carter KD , Fuss JM , Spencer AJ , Hodge CP . Caries experience in existing and new nursing home residents in Adelaide, Australia. Gerodontology. 2002;19(1):30‐40. 12164237 10.1111/j.1741-2358.2002.00030.x

[15] Maupome G , Wyatt CC , Williams PM , Aickin M , Gullion CM . Oral disorders in institution‐dwelling elderly adults: a graphic representation. Spec Care Dentist. 2002;22(5):194‐200. 12580358 10.1111/j.1754-4505.2002.tb00270.x

[16] Shimazaki Y , Soh I , Koga T , Miyazaki H , Takehara T . Relationship between dental care and oral health in institutionalized elderly people in Japan. J Oral Rehabil. 2004;31(9):837‐842. 15369462 10.1111/j.1365-2842.2004.01320.x

[17] Wyatt CC . Elderly Canadians residing in long‐term care hospitals: part I. Medical and dental status. J Can Dent Assoc. 2002;68(6):353‐358. 12034071

[18] Ghanbari‐Jahromi M , Bastani P , Jalali FS , Delavari S . Factors affecting oral and dental services; utilization among Elderly: a scoping review. BMC Oral Health. 2023;23(1):597. 37635217 10.1186/s12903-023-03285-4 PMC10464329

[19] Dolan TA , Atchison K , Huynh TN . Access to dental care among older adults in the United States. J Dent Educ. 2005;69(9):961‐974. 16141082

[20] Association AD . ADA Policy on Teledentistry; 2020.

[21] Ben‐Omran MO , Livinski AA , Kopycka‐Kedzierawski DT , et al. The use of teledentistry in facilitating oral health for older adults: a scoping review. J Am Dent Assoc. 2021;152(12):998‐1011. 34521539 10.1016/j.adaj.2021.06.005 PMC8627480

[22] Glassman P , Harrington M , Mertz E , Namakian M . The virtual dental home: implications for policy and strategy. J Calif Dent Assoc. 2012;40(7):605‐611. 22916382 PMC3477859

[23] Gibson G , Wehler CJ , Jurasic MM . Providing effective dental care for an ageing population. Int Dent J. 2022;72(4S):S39‐S43. 36031324 10.1016/j.identj.2022.06.011 PMC9437804

[24] Gurgel‐Juarez N , Torres‐Pereira C , Haddad AE , et al. Accuracy and effectiveness of teledentistry: a systematic review of systematic reviews. Evid Based Dent. 2022:1‐8. 10.1038/s41432-022-0257-8 PMC9264296 35804195

[25] Mariño RTU , Marwaha P , Collmann R , et al. Teleconsultation/telediagnosis using teledentistry technology: a pilot feasibility study. International Journal on Advances in Life Sciences. 2014;6:291‐299.

[26] Queyroux A , Saricassapian B , Herzog D , et al. Accuracy of teledentistry for diagnosing dental pathology using direct examination as a gold standard: results of the Tel‐e‐dent Study of Older Adults Living in Nursing Homes. J Am Med Dir Assoc. 2017;18(6):528‐532. 28236609 10.1016/j.jamda.2016.12.082

[27] Tynan A , Deeth L , McKenzie D , et al. Integrated approach to oral health in aged care facilities using oral health practitioners and teledentistry in rural Queensland. Aust J Rural Health. 2018. 10.1111/ajr.12410 29660771

[28] Tynan A , Deeth L , McKenzie D . An integrated oral health program for rural residential aged care facilities: a mixed methods comparative study. BMC Health Serv Res. 2018;18(1):515. 29970073 10.1186/s12913-018-3321-5 PMC6029389

[29] Marino R , Tonmukayakul U , Manton D , Stranieri A , Clarke K . Cost‐analysis of teledentistry in residential aged care facilities. J Telemed Telecare. 2016;22(6):326‐232. 26429795 10.1177/1357633X15608991

[30] Ghezzi EM , Niessen LC , Jones JA . Innovations in geriatric oral health care. Clin Geriatr Med. 2023;39(2):343‐357. 37045537 10.1016/j.cger.2023.01.005

[31] Hartshorn JE , Nair RU . Dental innovations which will influence the oral health care of baby boomers. Spec Care Dentist. 2023;43(3):359‐369. 36782274 10.1111/scd.12835

[32] Oishi MM , Childs CA , Gluch JI , Marchini L . Delivery and financing of oral health care in long‐term services and supports: a scoping review. J Am Dent Assoc. 2021;152(3):215‐223. 33632411 10.1016/j.adaj.2020.12.004

[33] Harris PA , Taylor R , Thielke R , et al. Research electronic data capture (REDCap)–a metadata‐driven methodology and workflow process for providing translational research informatics support. J Biomed Inform. 2009;42(2):377‐381. 18929686 10.1016/j.jbi.2008.08.010 PMC2700030

[34] Corp I . IBM SPSS Statistics for Windows. 2021. 28.0 ed.

[35] Team RC . R: a Language and Environment for Statistical Computing. R Foundation for Statistical Computing; 2024.

[36] Tomuro K . Development of oral home telecare programme for the home‐dwelling elderly: a pilot study. Gerodontology. 2004;21(3):177‐180. 15369021 10.1111/j.1741-2358.2004.00021.x

[37] Hartshorn JE , Cowen HJ , Comnick CL . Cluster randomized control trial of nursing home residents' oral hygiene following the Mouth Care Matters education program for certified nursing assistants. Spec Care Dentist. 2021;41(3):372‐380. 33587781 10.1111/scd.12577 PMC8248067

[38] Scannapieco FA , Amin S , Salme M , Tezal M . Factors associated with utilization of dental services in a long‐term care facility: a descriptive cross‐sectional study. Spec Care Dentist. 2017;37(2):78‐84. 27763678 10.1111/scd.12208

[39] Czwikla J , Rothgang H , Schwendicke F , Hoffmann F . Dental care utilization among home care recipients, nursing home residents, and older adults not in need of long‐term care: an observational study based on German insurance claims data. J Dent. 2023;136:104627. 37473830 10.1016/j.jdent.2023.104627

[40] Willems MS , Hollaar VRY , van der Maarel‐Wierink CD , van der Putten GJ , Satink T . Care‐resistant behaviour during oral examination in Dutch nursing home residents with dementia. Gerodontology. 2023;40(3):299‐307. 36000466 10.1111/ger.12654

[41] Chamut S , Shoff C , Yao K , Fleisher LA , Chalmers NI . Oral health among Medicare beneficiaries in nursing homes. JAMA Netw Open. 2023;6(9):e2333367. 37698864 10.1001/jamanetworkopen.2023.33367 PMC10498323

[42] Estai M , Bunt S , Kanagasingam Y , Kruger E , Tennant M . Diagnostic accuracy of teledentistry in the detection of dental caries: a systematic review. J Evid Based Dent Pract. 2016;16(3):161‐172. 27855831 10.1016/j.jebdp.2016.08.003

[43] Flores A , Lazaro SA , Molina‐Bastos CG , et al. Teledentistry in the diagnosis of oral lesions: a systematic review of the literature. J Am Med Inform Assoc. 2020;27(7):1166‐1172. 32568392 10.1093/jamia/ocaa069 PMC7647318

[44] Pandey P , Jasrasaria N , Bains R , et al. The efficacy of dental caries telediagnosis using smartphone: a diagnostic study in geriatric patients. Cureus. 2023;15(1):e33256. 36741615 10.7759/cureus.33256 PMC9891317

[45] Estai M , Kanagasingam Y , Huang B , et al. The efficacy of remote screening for dental caries by mid‐level dental providers using a mobile teledentistry model. Community Dent Oral Epidemiol. 2016;44(5):435‐441. 27111291 10.1111/cdoe.12232

[46] Kelly MC , Caplan DJ , Bern‐Klug M , et al. Preventive dental care among Medicaid‐enrolled senior adults: from community to nursing facility residence. J Public Health Dent. 2018;78(1):86‐92. 28884829 10.1111/jphd.12247

[47] Almomani FM , Bani‐Issa W . Physical, mental and cognitive disabilities in relation to utilization of dental care services by nursing home residents. Spec Care Dentist. 2017;37(3):126‐133. 28140479 10.1111/scd.12216

[48] Dye BA , Fisher MA , Yellowitz JA , Fryar CD , Vargas CM . Receipt of dental care, dental status and workforce in U.S. nursing homes: 1997 National Nursing Home Survey. Spec Care Dentist. 2007;27(5):177‐186. 17990476 10.1111/j.1754-4505.2007.tb00343.x

[49] Rawal K . End of Life Dental Service Utilization by Geriatric Patients in a Long‐term Care Setting. Boston University Theses & Dissertations; 2018.

